# The Role of Sex in the Risk of Mortality From COVID-19 Amongst Adult Patients: A Systematic Review

**DOI:** 10.7759/cureus.10114

**Published:** 2020-08-29

**Authors:** Monica Kelada, Ailin Anto, Karishma Dave, Sohag N Saleh

**Affiliations:** 1 Infectious Diseases, Imperial College London, London, GBR; 2 Pharmacology, Imperial College London, London, GBR

**Keywords:** covid-19, sex, gender, mortality, review

## Abstract

A worldwide outbreak of coronavirus disease 2019 (COVID-19), identified as being caused by the severe acute respiratory syndrome coronavirus 2 (SARS-COV-2), was classified as a Public Health Emergency of International Concern by the World Health Organisation (WHO) on January 30, 2020. Initial sex-disaggregated mortality data emerging from the Wuhan province of China identified male sex as a risk factor for increased COVID-19 mortality.

In this systematic review, we aimed to assess the role of sex in the risk of mortality from COVID-19 in adult patients through comparison of clinical markers and inflammatory indexes.

A systematic search was conducted on the following databases: PubMed, WHO COVID-19 database, Ovid MEDLINE, and Web of Science between the dates of June 15, 2020, and June 30, 2020. Key search terms used included: “sex”, “gender”, “SARS-COV-2”, “COVID” and “mortality”. We accepted the following types of studies concerning adult COVID-19 patients: retrospective cohort, observational cohort, case series, and applied research. Further studies were extracted from reference searching. The risk of bias was determined using the National Institutes of Health Quality Assessment Tool for Observational Cohort, Cross-Sectional Studies, and Case Series.

We identified a total of 16 studies published between January 2020 and June 2020 for analysis in this systematic review. Our study population consisted of 11 cohort studies, four case series, and one genetic study, including a total of 76,555 participants. Ten of the studies included in this review observed a higher risk of mortality among males compared to females, and eight of these studies found this risk to be statistically significant.

Sex-disaggregated COVID-19 mortality data identifies male patients with comorbidities as being at an increased risk of mortality worldwide. Further investigation revealed differences in immune response regulated by sex hormones, angiotensin-converting enzyme 2 (ACE2) expression, and health behaviours as contributing factors to increased risk of mortality from COVID-19 among males.

Nine out of the 16 studies included were conducted in China. In order to comprehensively assess sex-differences in the risk of mortality from COVID-19, more studies will need to be conducted worldwide. Sex-disaggregated COVID-19 data published in the medical literature is limited, however it has become evident that male sex is an important risk factor for mortality. Further exploration into the impact of sex on this pandemic is required in order to develop targeted therapies, as well as public health policies, and to prevent sex bias in treatment.

## Introduction and background

In December 2019, a rise in pneumonia cases of unknown aetiology was seen in the Wuhan province of China. Approximately one month later, the coronavirus disease 19 (COVID-19), caused by severe acute respiratory syndrome coronavirus 2 (SARS-CoV-2), was identified as the pathogenic source of the disease [1]. The virus is transmitted by talking, coughing, sneezing, aerosols and is now thought to be airborne [2]. The rapidly spreading and contagious nature of the virus led to a Public Health Emergency of International Concern being declared by the World Health Organisation (WHO) as of January 30, 2020 [3].   

Mild illness may present with symptoms such as fever, malaise, headache, muscle pain, dry cough, and sore throat. However, more severe disease may progress to Acute Respiratory Distress Syndrome (ARDS) and death [3].  

As of June 30, 2020, England had 39,177 COVID-19 fatalities of which 56.9% were males and 43.1% were females [4]. Further exploration into the impact of sex on this pandemic is required to develop targeted therapies and public health policies. Sex-disaggregated analysis of the COVID-19 outbreak is important, as mortality data from 49 countries indicate that males have a higher overall mortality rate than females [4]. Although male and female susceptibility is the same, it has become evident that the male sex is an important risk factor for mortality [5].

In this systematic review we aimed to assess the role of sex in the risk of mortality from COVID-19 in adult patients through comparison of clinical markers and inflammatory indexes.

## Review

Methodology

Search Strategy   

A systematic search was conducted on the following electronic databases: PubMed, WHO COVID-19 database, Ovid MEDLINE and Web of Science between the dates of June 15, 2020, and June 30, 2020. This systematic review was conducted in line with the Preferred Reporting Items for Systematic Reviews and Meta-Analyses (PRISMA) guidelines  and has been registered on PROSPERO (identifier number: CRD42020196076) [6].  

A search strategy containing keywords was implemented across all search domains. Our search strategy consists of the following keywords: “male”, “female”, “men”, “women”, “sex”, “gender”, “corona”, “COVID-19”, “Cov2”, “SARS”, “SARS-COV-2”, “SARS-2”, “SARS-corona”, “severe acute respiratory syndrome”, “hormone”, “androgen”, “testosterone”, “oestrogen”, “estrogen”, “oestradiol”, “estradiol”, “ACE2”, “ACE-2”, “TMPRSS2”, “mortality”, “morbidity”, “death”.   One example of a search string used is: “((COVID-19) OR (Coronavirus)) AND ((Gender) OR (sex) OR (male) OR (female)) AND ((mortality) OR (death rate))”.  

Selection Criteria  

The preliminary search yielded 1092 papers. After removal of duplicates, 655 abstracts and titles were screened for relevancy, through which 598 papers were excluded. The remaining 57 papers underwent full text screening. An additional 25 papers were retrieved through screening of relevant references. A total of 82 papers underwent full text screening. After applying inclusion and exclusion criteria, 16 papers were selected for analysis in this systematic review.   

We accepted the following types of studies: retrospective cohort, observational cohort, case series, and applied research. Papers from any country concerning confirmed COVID-19 adult patients were accepted if they were written in English.  Papers concerning paediatric (being under 18 years of age) and pregnant patients were excluded. Papers addressing other viral respiratory diseases, such as severe acute respiratory syndrome (SARS) and Middle East respiratory syndrome (MERS), were also excluded.  

Our outcome of interest was mortality rate of males and females diagnosed with COVID-19. Indicators that we regarded as potential reasons for the difference in mortality included: immunoglobulin G (IgG) antibody levels, immune cell levels, inflammatory indexes, receptor expression, hormone levels, behavioural factors , and disease severity.  

**Figure 1:**
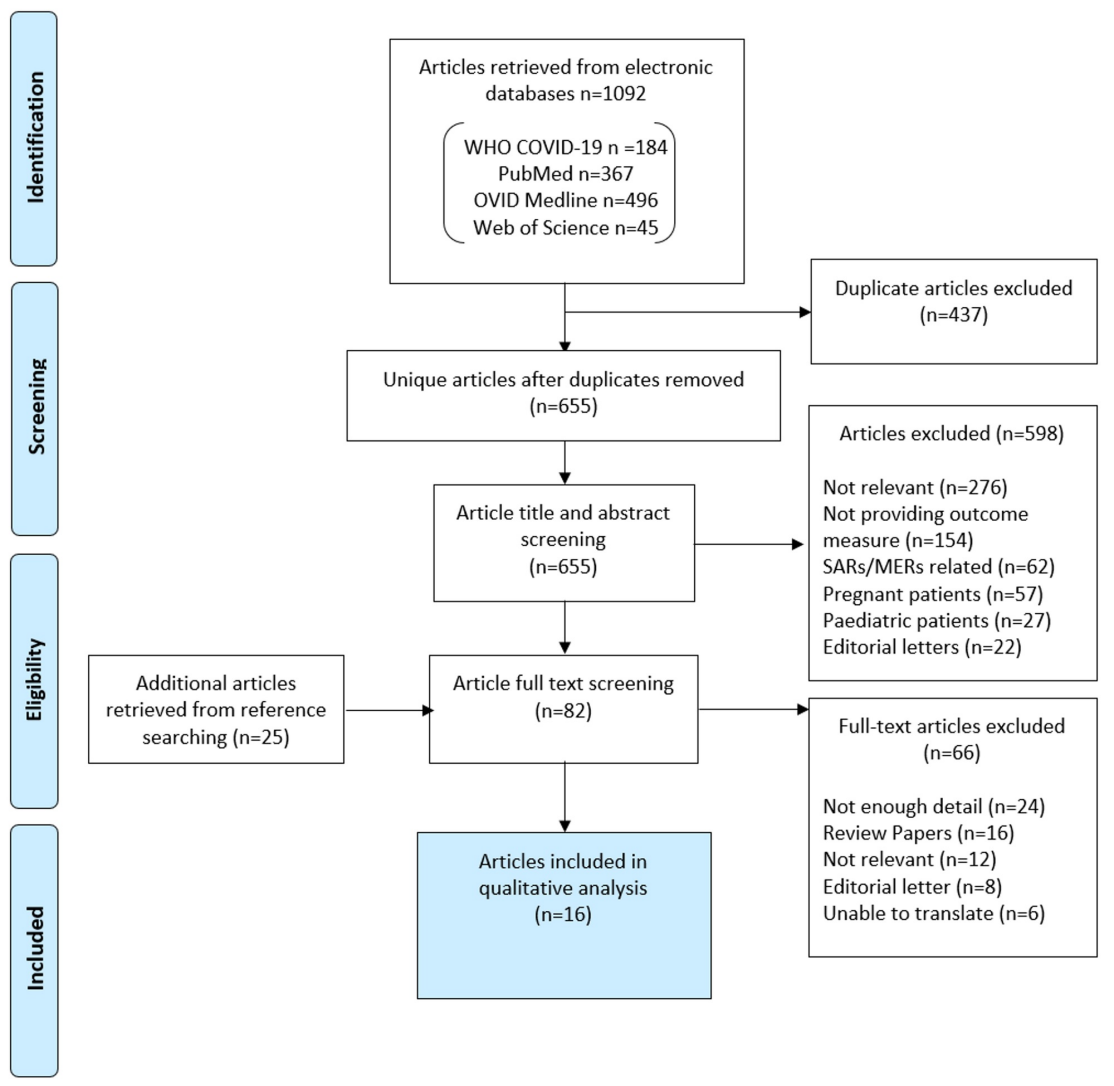
PRISMA flow diagram PRISMA flow diagram to show study identification, screening, inclusion and exclusion against specific pre-defined criteria. COVID-19: coronavirus disease 2019, WHO: World Health Organization, SARS: severe acute respiratory syndrome, MERS: Middle East respiratory syndrome

Two reviewers screened the abstracts and titles retrieved from the searches. The third reviewer conducted a final check on the relevance of the papers against the inclusion and exclusion criteria. Data retrieved from the studies were collated into tabular form outlining the study design and main findings.  Figure 1 shows the PRISMA flow diagram detailing the study identification, screening, and selection process. 

Risk of Bias Assessment  

Risk of bias was assessed using the National Institutes of Health (NIH) Quality Assessment Tool for Cohort, Cross-Sectional Studies and Case series [7]. Two review authors assessed the risk of bias and this was reviewed by the third author.

Results

We identified a total of 16 studies published between January 2020 and June 2020 for analysis in this systematic review. Our study population consisted of 11 cohort studies, four case series and one genetic study, including a total of 76,555 participants. The characteristics and main outcomes of each study are summarised in Table 1.  

**Table 1:** Sixteen inclusion studies detailing study country, type, population, purpose and main outcomes. SARS-CoV-2: severe acute respiratory syndrome coronavirus 2, COVID-19: coronavirus disease 2019, ACE2: angiotensin-converting enzyme 2, IgG: immunoglobulin G, OR: odds ratio

Reference	Country	Study Design	Sample Population	% Males	% Females	Study Purpose	Outcome/Conclusions
Qin L. et al (2020) [8]	China	Retrospective Cohort Study	548 COVID-19 inpatients	50.9	49.1	To investigate how the effect of sex on inflammation affects COVID-19 outcomes	Mortality was higher in males (22.2% vs 10.4%). Relative Risk for mortality was 2.2 for males compared to females. Males had higher rates of lymphopenia, thrombocytopenia. Multiple linear regression method revealed greater levels of inflammation indexes in males.
Asfahan S. et al (2020) [9]	China	Retrospective Cohort study	44,672 COVID-19 inpatients	51.4	48.6	To describe mortality characteristics for COVID-19 from publicly available data from China	81% of deaths were in the ages above 60 years, 63.8% of deaths were males. Logistic regression risk factors showed that only age and comorbidities significantly affected mortality. Sex and occupation when adjusted for other factors were not significant predictors of mortality - although case fatality rate was higher in males (2.8%) compared to females (1.7%).
Borobia AM. et al (2020) [10]	Spain	Retrospective Cohort Study	2226 COVID-19 in-patients	48.2	51.8	To describe the clinical characteristics of hospitalized patients with COVID-19	Mortality was higher in males than females (26.6% vs. 15.1%). Compared with the entire cohort, the patients admitted to the ICU had a higher male/female ratio (3.2 vs. 0.93). In multivariate logistic regression model, male sex was associated with higher probability of death from COVID-19.
Yang X. et al (2020) [11]	China	Retrospective Cohort Study	52 critically ill COVID-19 adults	67	33	To describe the clinical course and outcome of critically ill patients with COVID-19	67% of critically ill patients were males. (Critically ill patients were defined as those admitted to the Intensive Care Unit (ICU) who required mechanical ventilation or had a fraction of inspired oxygen of at least 60% or more)
Li X. et al (2020) [12]	China	Retrospective Cohort Study	269 severe COVID-19 inpatients	56.9	43.1	To evaluate the severity on admission, complications and outcomes of COVID-19 patients	Multivariable Cox proportional hazards regression analysis showed that male sex is a risk factor (adjusted HR, 1.7; 95% CI, 1.0-2.8). Other risk factors identified: Older age, leukocytosis, hyperglycaemia, high lactate dehydrogenase, high corticosteroid dose, cardiac injury.
Shi Y.et al (2020) [13]	China	Retrospective Cohort study	49 severe COVID-19 inpatients	73.5	26.5	To explore host risk factors. To establish a score system to identify high risk patients	Out of the 49 severe patients, 36 were male and 13 were female. There were significantly more males among the severe cases (P=0.003). On multivariate analysis, male sex is associated with severe disease at admission (OR 3.68 [95% CI 1.75–7.75], P = 0.001).
Liu Y. (2020) [14]	China	Retrospective Cohort study	245 COVID-19 patients	46.5	53.5	To investigate whether neutrophil-to-lymphocyte ratio (NLR) can serve as a predictor of hospital mortality	The odd ratio for mortality was 1.10 in males (CI 1.02-1.10; P=0.016). Sensitivity analysis was used to convert NLR into a categorical tertile variable. Males made up 61% of tertile 3 (most severe tertile). NLR is an independent risk factor for mortality especially in males.
Jin J. et al (2020) [5]	China	Retrospective Cohort study	43 case series hospitalised patients	51.2	48.8	To compare the severity and mortality between male and female patients with COVID-19	Males and females had the same susceptibility. Male cases were generally more serious than female (P = 0.035). Males were more prone to dying (χ2 test, P = 0.016). 37 patients died, 70.3% were male, 29.7% were female.
			37 cases from public data set of the first patients who died of COVID-19 and 1019 who survived	50.8	49.2		The number of males who died from COVID-19 is 2.4 times that of females (70.3 vs. 29.7%, P = 0.016).
Zeng F. et al (2020) [15]	China	Retrospective Cohort study	331 COVID-19 inpatients	38.4	61.6	To investigate if there is a difference in serum IgG antibody between males and females	IgG antibody tended to be stronger in female patients in the early phase of infection. In female patients, the concentration of COVID-19 IgG antibody continuously increased from mild to severe status and then decreased in recovering patients. In male patients, the IgG antibody rose from mild to general status and then decreased from general status patients to recovering patients. Females patients generated a high level of COVID-19 IgG antibody relative to male patients in severe status. Female level was higher in week 2-4 but after week 4 there was no longer a difference between the sexes.
Li M. et al (2020) [16]	China	Retrospective Cohort study	31 GTEx normal tissues	N/A	N/A	To investigate the difference in ACE2 expression in different human tissue to understand COVID-19 mechanism of infection	No significant difference found in ACE2 expression between males and females. Negative correlation found between ACE2 and CD8^+^ cells, interferon response and B cells in female lungs. Positive correlation found between ACE2 and CD8^+^, interferon response and B cells in male lungs.
Docherty A. et al (2020) [17]	UK	Observational Cohort Study	20,133 COVID in-patients	60	40	To characterise the clinical features of patients admitted to hospital with COVID-19 and explore risk factors associated with mortality. Independent risk factors for mortality were increasing age, male sex, and chronic comorbidity, including obesity.	Multivariable Cox proportional hazards ratio model: Female sex was associated with lower mortality (0.81 hazard ratio, CI (95%) 0.75 to 0.86, P<0.001).
Fagone P. et al (2020) [18]	Italy	Genetic Study	134 healthy lung tissue samples	70	30	Aimed to identify a specific gene signature characterising SARS-COV-2 infection, employing gene term enrichment analysis on the differentially expressed genes.	Gene term enrichment analysis for the 94 upregulated genes identified three significantly altered pathways upon SARS-CoV-2 infection: “cytokine-mediated signalling pathway”, “IL-17 signalling pathway”, and “defence response to other organism”. Comparison of transcriptomic profile of lung tissue from healthy women and men revealed female lung tissues has a more similar phenotype to that induced upon SARS-CoV-2 infection than identical tissues from men.
Rastrelli G. et al (2020) [19]	Italy	Case Series	31 male COVID-19 inpatients recovered in Respiratory Intensive care [RICU]).	100	0	To estimate the association between Testosterone level and COVID-19 clinical outcomes (outlined as conditions requiring transfer to higher or lower intensity of care).	Total Testosterone (TT) and calculated Free Testosterone (cFT) showed a significant decline according to worsening outcomes. Linear regressions showed that for each nmol/L decrease in TT and 10 pmol/L decrease in cFT, the probability of worse clinical outcomes increased [T (p= 0.017), cFT (p=0.007)]. Lower baseline levels of TT and cFT levels predict poor mortality in COVID-19 infected males admitted to Respiratory Intensive Care Unit (RICU).
Richardson S. et al (2020) [20]	USA	Case Series	5700 COVID-19 in-patients	60.3	39.7	To describe the clinical characteristics and outcomes of patients with COVID-19.	Mortality rates were higher for male compared with female patients at every 10-year age interval older than 20 years. Mortality was 0% (0/20) for male and female patients younger than 20 years.
Suleyman G. et al (2020) [21]	USA	Case Series	463 COVID-19 in-patients	44.1	55.9	To describe clinical characteristics and outcomes of COVID-19 patients. To perform a comparative analysis of hospitalised and ambulatory patient populations	Male sex was independently associated with ICU admission (OR= 2.0, P=0.006) and significantly associated with mortality (OR=1.8, P= 0,03). Multivariable analysis of patient characteristics and need for mechanical ventilation in ICU showed high association with male sex (OR=2.9, P<0.001).
Grasselli G. et al (2020) [22]	Italy	Case Series	1591 COVID-19 in-patients admitted to ICU	82	18	To characterise patients requiring ICU admission from COVID-19	The majority of critically ill patients admitted to ICU were older men. 82% of COVID-19 inpatients admitted to ICU from February 20^th ^to March 8^th^ 2020 were male. The median age of patients admitted to ICU was 63 years old.

Sex and Mortality From COVID-19  

**Figure 2:**
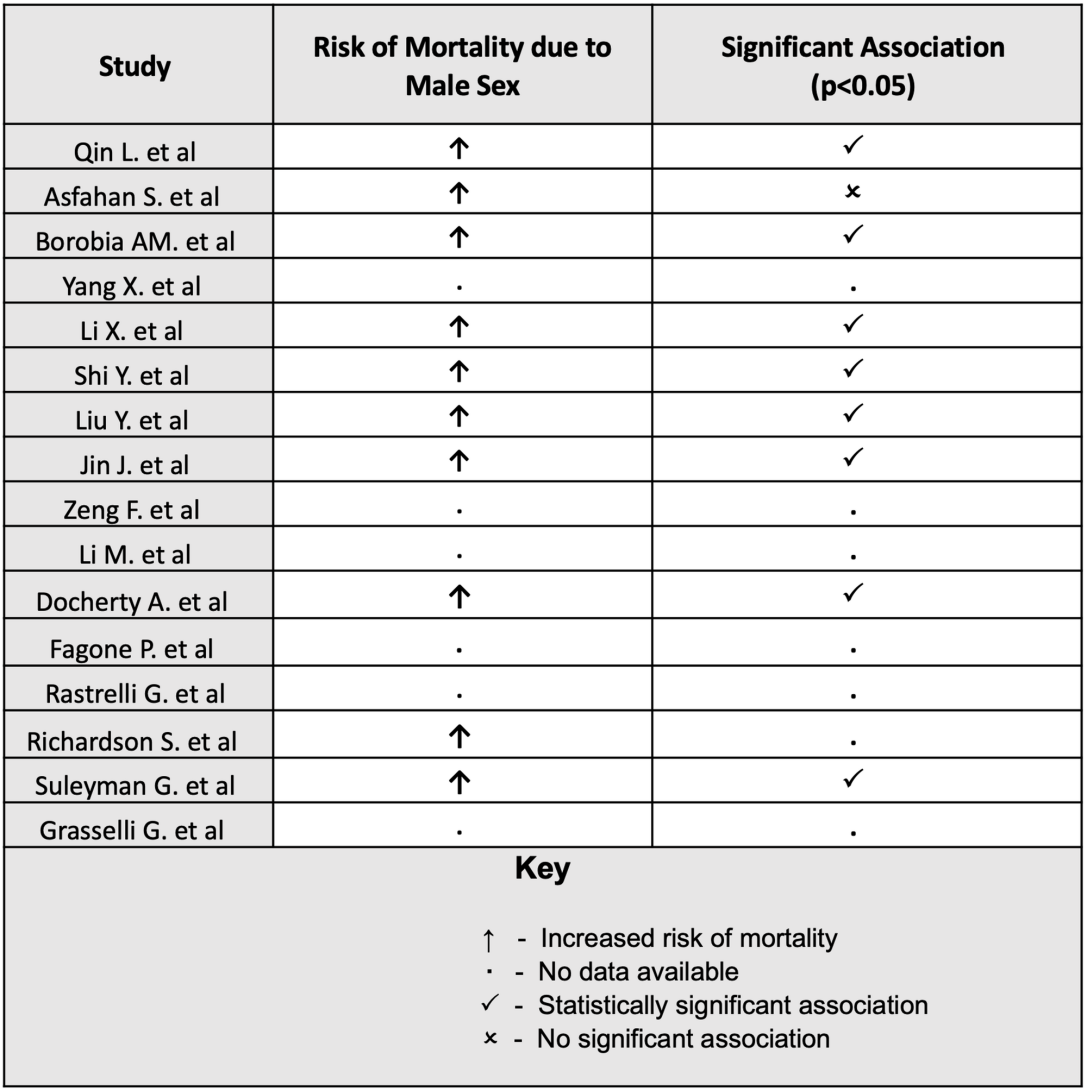
Male sex and mortality from COVID-19 This figure shows which of the included studies observed an increased risk of mortality due to male sex, and which studies observed a significant association (p<0.05).

Ten studies from the database search observed higher risk of mortality amongst males compared to females. Eight studies found male sex to be significantly associated with increased risk of mortality from COVID-19. One study found no significant association between male sex and mortality after adjusting for confounders. Figure 2 indicates which studies have observed an increased risk of mortality in males and those in which this association is significant.  

Risk of Bias Assessment Results

Table 2 summarises the risk of bias results assessed using the NIH quality assessment tool [7].

**Table 2:** Results of the National Institutes of Health Quality Assessment Tool for Cohort, Cross-Sectional Studies and Case series [7] for the 16 inclusion studies. Cohort and Cross-Sectional Studies: 1. Was the research question or objective in this paper clearly stated? 2. Was the study population clearly specified and defined? 3. Was the participation rate of eligible persons at least 50%? 4. Were all the subjects selected or recruited from the same or similar populations? Were inclusion and exclusion criteria for being in the study prespecified and applied uniformly? 5. Was a sample size justification, power description, or variance and effect estimates provided? 6. Were the exposure(s) of interest measured prior to the outcome(s) being measured? 7. Was the timeframe sufficient so that one could reasonably expect to see an association between exposure and outcome if it existed? 8. For exposures that can vary in amount or level, did the study examine different levels of the exposure as related to the outcome? 9. Were the exposure measures (independent variables) clearly defined, valid, reliable, and implemented consistently? 10. Was the exposure(s) assessed more than once over time? 11. Were the outcome measures (dependent variables) clearly defined, valid, reliable, and implemented consistently across all study participants? 12. Were the outcome assessors blinded to the exposure status of participants? 13. Was loss to follow-up after baseline 20% or less? 14. Were key potential confounding variables measured and adjusted statistically for their impact on the relationship between exposure(s) and outcome(s)? Case Series: 1. Was the study question or objective clearly stated? 2. Was the study population clearly and fully described, including a case definition? 3. Were the cases consecutive? 4. Were the subjects comparable? 5. Was the intervention clearly described? 6. Were the outcome measures clearly defined, valid, reliable, and implemented consistently across all study participants? 7. Was the length of follow-up adequate? 8. Were the statistical methods well-described? 9. Were the results well-described?

NIH Risk of Bias Assessment														
Cohort and Cross-Sectional Studies														
Reference	1	2	3	4	5	6	7	8	9	10	11	12	13	14
Qin L. et al	Y	Y	NA	Y	N	Y	Y	NA	Y	Y	Y	NA	NR	Y
Asfahan S. et al	Y	N	NA	Y	NA	Y	NR	N	Y	NA	Y	NA	NA	Y
Borobia AM. et al	Y	Y	NA	Y	N	Y	Y	Y	Y	N	Y	NA	NR	Y
Yang X. et al	Y	Y	NA	Y	Y	Y	Y	Y	Y	N	Y	NA	NR	Y
Li X. et al	Y	Y	NA	Y	Y	Y	Y	Y	Y	NA	Y	NA	NR	Y
Shi Y. et al	Y	Y	NA	Y	N	Y	NR	N	N	NA	N	NA	NA	N
Liu Y. et al	Y	Y	Y	Y	Y	Y	Y	Y	Y	N	N	NA	N	Y
Jin J. et al	Y	N	NA	N	N	Y	NA	Y	N	N	Y	NA	NA	Y
Zeng F. et al	Y	Y	NA	Y	N	N	NR	Y	N	NA	Y	NA	NA	NR
Li M. et al	Y	Y	NA	NR	N	N	NR	NA	Y	N	Y	NA	NA	Y
Docherty A. et al	Y	Y	NA	N	Y	Y	Y	Y	Y	N	Y	NA	NR	Y
Fagone P. et al	Y	Y	NA	NA	N	Y	NA	NA	Y	NA	Y	NA	NA	NR
Case Series														
Reference	1	2	3	4	5	6	7	8	9					
Rastrelli G. et al	Y	Y	Y	Y	NA	Y	Y	Y	Y					
Richardson S. et al	Y	Y	Y	Y	NA	Y	Y	Y	Y					
Suleyman G. et al	Y	Y	Y	Y	NA	Y	Y	Y	Y					
Grasselli G. et al	Y	Y	Y	Y	Y	Y	N	Y	Y					
Key: Y = Yes, N = No, NA = Not applicable, NR = Not reported														

Discussion

We undertook a comprehensive search of the literature concerning sex-disaggregated mortality from COVID-19 in order to summarise the potential underlying reasons for the disparity in mortality rate. We found that published evidence suggests the female sex has a protective role against COVID-19 mortality. Main reasons for this finding include the higher levels of the circulating form of ACE2 in females, the immunostimulatory effect of female sex hormones, more rapid clearance of pathogens by the female immune system and females tending to display disease-preventing behaviours.  

Sex Differences in ACE2 Expression

Differences in angiotensin-Converting Enzyme 2 (ACE2) expression between sexes is thought to contribute to the higher male mortality rate. ACE2 degrades Angiotensin II into Angiotensin 1-7, counteracting the Renin-Angiotensin-System (RAS) axis. This reduces the effects of the RAS axis which usually increases blood pressure, sympathetic tone, vasoconstriction, inflammation, and fibrosis. ACE2 also serves as the primary receptor for SARS-CoV-2 cellular invasion [23]. The viral spike protein contains the S1 domain, which serves as a receptor-binding portion, and the S2 domain which facilitates cellular-viral fusion [24,25]. The high affinity of SARS-CoV-2 for the ACE2 receptor facilitates viral spread between person to person [26]. It is also thought that SARS-CoV-2 infection downregulates ACE2 expression, reducing its protective role and explaining the progression of patients into ARDS [23].  

ACE2 is expressed on the PAR region of the X chromosome, which has a greater chance of escaping X chromosomal inactivation [27]. ACE2 is also upregulated by oestrogen leading to disparity in ACE2 expression in some organs between the sexes [27,28]. The paradox of ACE2 upregulation yet lower female mortality can be explained by a few theories. There are two types of ACE2: the membrane-bound, which provides the viral entry point, and the circulating which has a cardiovascular protective function. It is thought that females express more of the circulating ACE2 providing protection against disease progression into ARDS [29].   

Vikse et al. suggest that the testes may serve as a reservoir for SARS-CoV-2, delaying viral clearance and increasing the likelihood of systemic tissue damage. The high levels of ACE2 expression and the immune-privileged nature of this organ concur with this theory [30]. It is also thought that amino acid substitutions can influence viral S1-ACE2 interaction and viral infectivity. Due to hemizygosity in males, carrying a viral-boosting allelic variant of ACE2 may lead to increased susceptibility to severe disease [31,32]. Li et al. propose that increased male mortality can be attributed to the increased likelihood of a cytokine storm in the lungs, accelerating progression into ARDS. They found a positive correlation between ACE2 expression and immune cell levels (Natural Killer (NK) cells, CD8+ cells) in male lung tissue whereas the opposite was found in females [16].  

Role of Sex Hormones

Previous literature shows that differences in sex hormones impact the immune system and therefore may play a role in SARS-CoV-2 clearance. It is thought that testosterone (T) has both protective and adverse effects on mortality risk.   

Low levels of T appear to be linked with increased susceptibility of respiratory diseases [33]. Rastrelli et al. demonstrate that low levels of T and circulating free testosterone (cFT) are predictors for adverse outcomes and mortality from COVID-19 [19]. This concurs with existing literature in which an association between hypogonadism and proinflammatory cytokine levels is observed [33,34]. Severe infections are also associated with a reduction in numbers of CD4^+^ T cells, CD8^+^ T cells, B cells, and NK cells. The presence of androgen receptors (AR) on these cells suggests that T is important in their function [34].  

In addition to this, T plays a complex role in coagulation which could affect male mortality rate. Intravascular thrombosis and endothelial dysfunction complicate COVID-19 prognosis. Published evidence indicates this occurs more frequently in males than females [35].  T augments activation and aggregation of platelets by increasing platelet expression of thromboxane A2 receptors [36].  In contrast, a negative correlation between serum T levels and platelet reactivity has been discovered by an ex vivo study [37]. T enhances the production of endothelial nitric oxide, a potent vasodilator and inhibitor of platelet recruitment. Mean platelet volume, a biological indicator of platelet activation, is seen to be increased in hypogonadal males [38]. Therefore, it could be hypothesised that T protects males against new thrombotic events in COVID-19, an effect that is lost through hypogonadism [33].   

T has a cardioprotective role and promotes myocardial health. Thus, males with hypogonadism are predisposed to increased cardiovascular risk from COVID-19. T is vital in regulating glucose and maintaining favourable lipid metabolism [35]. Furthermore, being a rapid onset vasodilator, T reduces blood pressure by blocking calcium channel opening. Males with cardiovascular diseases (CVD) tend to have low serum T levels [39]. This further illustrates the importance of T in protecting against chronic CVD, as well as acute cardiac injury, which is typically associated with severe COVID-19 disease [17,33,40].

Although hypogonadism appears to be a risk factor for mortality, a contradictory ‘Testosterone driven COVID-19’ theory exists [28]. Transmembrane Protease Serine 2 (TMPRSS2) cleaves the viral S protein at two sites allowing penetration changes on which viral entry into cells depends. It is thought that increased male mortality could be attributed to the androgen regulation of TMPRSS2. There is discourse in the literature as some papers find that there is no significant difference in TMPRSS2 expression in the lungs between the sexes [41]. However, other papers find that males have significantly higher (P=0.029) expression of TMPRSS2 at the pulmonary level which may lead to viral progression and poorer outcomes [32].  

Oestrogen (E) is thought to have a protective role against COVID-19 mortality in females. Lower female mortality could be attributed to E stimulating immune cell development, namely B cells, leading to humoral anti-viral responses. E receptors, present on various leukocytes induce pro-inflammatory cytokine production such as interleukin (IL)-12, tumor necrosis factor-alpha (TNF-alpha) and chemokine (C-C motif) ligand 2 (CCL2) [42]. The activated lymphocytes and alveolar macrophages increase type 1 and 2 interferon (IFN) production, reducing viral load.   

Scotland et al. suggest that E may also affect leukocyte function. They found that female mice have an increased number of resident T lymphocytes and that their tissue macrophages have a higher density of toll-like receptor (TLR), specifically TLR2 and TLR4; this allows rapid detection and elimination of pathogens [43]. Channappanavar et al. also demonstrate E’s protective role as they found oophorectomy or treating female mice with an ER antagonist resulted in increased mortality from SARS-CoV-1, whereas gonadectomy did not affect mortality [44]. This finding may also be applicable to SARS-Cov-2, providing a potential explanation for higher male COVID-19 mortality. 

Sex Differences in Immune Regulation 

A study conducted by Zeng et al. highlights that females produce more serum (SARS-CoV-2) IgG in comparison to males in severe disease status [15]. TLR7, a pattern recognition receptor, is expressed on the X chromosome and can bypass X chromosomal inactivation [42]. Female X chromosomal homozygosity results in a greater gene dosage and expression of TLR7, allowing for stronger antigen detection [45]. TLR7 presenting plasmacytoid dendritic cells in females produce more type 1 IFN following ligand stimulation when compared to males [45]. In the presence of TLR7, type 1 IFN enhances B cell-mediated immunoglobulin secretion as well as proliferation [46]. These biological processes provide an explanation for higher serum IgG in females.  

Gene term enrichment analysis of the genes upregulated in a SARS-CoV-2 infection in human lung epithelium identify the ‘cytokine-mediated signalling pathway’ as the most significantly altered pathway [18]. Qin et al. observe that COVID-19 disease severity is positively correlated with inflammation [7]. Following viral invasion of the lungs, aberrant release of inflammatory cytokines (soluble IL-2, IL-6, IL-8, IL-10) and proteins (LDH, ferritin, high-sensitivity CRP [hs-CRP]) damage the alveolar epithelial cell barrier causing oedema and hypoxia leading to ARDS [8,17]. Inflammatory indexes within this cohort are significantly higher in males. This is worth noting as mortality within this cohort was twice as likely in males and this could be as a result of the immunopathogenic damage caused by excess cytokine storms promoting acute lung injury [8].  

Gene term enrichment analysis may provide further explanation for these sex-based differences in cytokine expression. Several Differentially Expressed Genes (DEGs) identified upon SARS-CoV-2 infection of human lung epithelium are found to be modulated by sex hormones. Neutrophil chemotactic factor CXCL1 and dendritic cell chemotactic factor CCL20 are significant DEGs upregulated in SARS-CoV-2 infection. Both factors are regulated by AR, providing further evidence for the role of excess cytokine storms observed in males increasing mortality [18].   

SARS-CoV-2 infection is known to result in significant lymphocytopenia, the extent of which differs between the sexes [8,10,11,14]. Yang et al. observe lymphocytopenia in 80% of their most critically ill patients [11]. Qin et al. similarly note that male COVID-19 patients have a lower overall lymphocyte count compared to females when adjusting for age and comorbidity [8]. Additionally, previous research finds that females have higher CD4^+^ T cell counts than age-matched males, and after in vitro stimulation females produce higher numbers of activated CD4^+^ T cells [44]. The greater reserve of CD4^+^ lymphocytes, combined with lower risk of lymphocytopenia in SARS-CoV-2 infection, may potentially decrease the risk of mortality from COVID-19 in females. 

Sex Differences in Behaviour

Behavioural differences are also thought to contribute to the difference in COVID-19 mortality between sexes. Males tend to partake in higher-risk behaviours such as smoking and drinking alcohol; the WHO reports that 40% of males worldwide smoke compared to 9% of females [47]. Additionally, it is thought that females are more likely to follow hygiene and preventative routines [48].  

A study by Guan et al. finds an association between disease severity and smoking. Smokers make up a greater proportion of severe COVID-19 patients compared to non-severe patients (16.9% and 11.8%, respectively) [49]. However, it is worth noting that in this study sex is not taken into consideration. Bagone et al. observe an association between cigarette smoke and increased heme oxygenase-1 induction (HO-1) of lung fibroblasts in mice. HO-1 is thought to have anti-viral and cytoprotective properties [50]. This confounds the previous understanding of the relationship between smoking and SARS-CoV-2 infection. Therefore, further research and clarification are needed to determine the precise mechanisms of this relationship.   

Strengths and limitations

Our review has several strengths. The search strategy we implemented contained a comprehensive list of search words resulting in an in-depth analysis of available evidence. We adopted a pragmatic search strategy approach, appropriate for an ongoing pandemic setting, which aligned with PRISMA guidelines and WHO recommendations for rapidly reviewing evidence in the context of emergencies. Papers also underwent an extensive appraisal before being included in our review as two reviewers assessed potential studies while a third reviewer verified this. Although the risk of bias assessment showed most of the studies used had little or no bias, we found that disease severity was measured differently between studies. As a result, comparison of patients between studies must be done carefully. 

There are several limitations to our review. We limited our electronic database search due to the dynamic and rapidly evolving nature of the COVID-19 pandemic, therefore we included publications only in English between January 2020 and June 2020. Our search strategy was also focused solely on sex and COVID-19 mortality; no comparisons were made regarding the role of sex in the SARS and MERS outbreaks. Additionally, nine out of the 16 studies used were conducted in China. In order to comprehensively assess sex differences in the risk of mortality from COVID-19, more studies will need to be conducted worldwide.  

Sex-disaggregated COVID-19 data published in medical literature is limited however it has become evident that the male sex is an important risk factor for mortality. Further exploration into the impact of sex on this pandemic is required in order to develop targeted therapies, as well as public health policies, and to prevent sex bias in treatment.    

## Conclusions

In conclusion, data emerging worldwide suggests that the male sex has a significant role in increasing risk of COVID-19 mortality amongst adult patients. This association may be explained by the findings that males tend to have lower serum IgG antibody generation, decreased CD4^+ ^T cell reserves, and lower circulating ACE2 expression when compared to females.  Male sex is also found to be associated with increased disease severity upon hospital admission, higher rates of ICU admission, and increased clinical markers such as lymphopenia and inflammatory indexes. Conversely, female sex is found to play a significant role in lowering risk of mortality from COVID-19.
